# Spatial and temporal distribution of population in urban agglomerations changes in China

**DOI:** 10.1038/s41598-022-12274-6

**Published:** 2022-05-18

**Authors:** Yanming Lyu, Fangye Jiang

**Affiliations:** College of Architecture and Urban-Rural Planning, Sichuan Agricultural University, 288 Jianshe Road, Dujiangyan City, Chengdu City, Sichuan Province China

**Keywords:** Energy and society, Sustainability

## Abstract

The spatial distribution of the urban agglomeration population has changed increasingly in rapid urbanization. It affects the urban economy, environment, transportation, and so on. Therefore, it is of great significance to understand the changes in the spatial distribution of population in urban agglomerations. This study used methods such as population center of gravity migration and Gini coefficient to explore changes in the spatial distribution of urban populations. The study found that the population center of the Beijing-Tianjin-Hebei urban agglomeration first migrated toward Beijing and then away from Beijing. During this process, the Gini coefficient increased from 0.62 to 0.64 and then decreased to 0.58, indicating that the population balance of the urban agglomeration firstly increased and then decreased. This result is consistent with the conclusion that we have established a simulated urban agglomeration for theoretical derivation. That is: in the early stage of urban agglomeration development, the population migrated to the central city; as the population of the central city became saturated, the urban agglomeration developed to a mature stage, and the population began to migrate to the peripheral cities. In this process, the population distribution center of urban agglomeration gradually shifted from central towns to peripheral towns. The degree of equilibrium in the spatial distribution of population has undergone an inverted u-shaped transition from non-equilibrium to equilibrium.

## Introduction

The population size is the total number of permanent residents living in a specific area in a certain period. It is closely related to urbanization level^1,2^, urban economics^3^, urban technological innovation^4^, urban environment^5^, resources^6,7^, and transportation^8,9^. Population size can promote urban technological innovation and improve urban economic development^10–13^. However, excessive population growth may also lead to environmental pollution^14^, resource overconsumption^7^, traffic congestion^15,16^, social-economic inequality^14,17^, urban form, and structure constitute inequality^18^. Thus, it is necessary to pay more attention to urbanization's changing urban population size.

The changing process of urban population size generally includes two periods^1^. At the first stage, the scale of the urban population increased significantly. At the second stage, the urban population tends to saturate and grow slowly^19^. Athens, Greece is in line with this law of population size changes t, from 1950 to 2000, it had been a linear growth, and the growth rate was relatively high, from 2001 to 2020, the development of the permanent population tends to be slow^19^. It shows a prominent characteristic of two-stage change.

Urbanization, economic development, public service level, life quality, and preferential local policies have impacted urban population growth^2,20^. In recent years, the urban spatial structures and economic characteristics have taken noticeable changes^21^, influencing the urban choice intention of residents and enterprises^2,22^. After satisfying basic needs, people pursued high-quality public services, excellent infrastructure, quality of life, and more robust local preferential policies. These factors have also affected the direction and speed of urban population migration^23^. Taken fast-growing China for example, the population of surrounding cities has rapidly flowed into megacities, resulting in many social problems (e.g. Traffic congestion, environmental pollution, high house price), and the population of the megacities is approaching saturation. In recent years, China's population transfer to small and medium-sized cities has increased, which has driven the spatial flow and exchange of factors such as social economy, urban transportation, regional culture and other elements in small and medium-sized cities and play a vital role in accelerating economic development, promoting urbanization and industrialization, and optimizing the geographical allocation of labor resources^22^.

It is also found that the dynamic change process of urban population size appears the characteristics of group development. In urban population growth and rapid economic development, cities are adjacent in space and cooperate in function, thus forming urban agglomeration^24,25^. In urban agglomerations, there are usually one or more central cities with a higher level of economic development and several peripheral towns with a relatively low level of economic growth^26^. More individuals pour into the central cities in urban agglomeration development due to their higher developed economy, modernization, pursuing high-density, multi-purpose development, and energy-saving transportation systems. The accumulation of the population in major cities promotes trade and economic development, with positive externalities, including improved division of labor, improving public facilities^10^, and increasing employment opportunities^27^. However, during urban agglomeration development, it has also led to more urban problems^6^, including the increase in population density^28^, urban congestion^29^, high living costs, and environmental deterioration^29^. More and more people are migrating to surrounding cities^6^, and "sleeping cities" have emerged in some places where residents sleep in one place at night and work in different urban areas during the day)^1^.

The above discussed the general change process of population size of cities and urban agglomerations. However, there are few studies on the fundamental laws of the population in urban agglomerations^1^. Failure to fully understand the mechanism of population change in urban agglomerations is likely to lead to ineffective government control of excessive population migration. If this situation continues, it may lead to economic, environmental, transportation, and other social problems. Therefore, it is necessary to explore the fundamental laws of population changes in urban agglomerations, reveal the mechanism of population migration, reasonably predict the direction of population migration in urban agglomerations, and provide corresponding suggestions and countermeasures for urban agglomerations and cities at different levels of development. The remainder of this paper is carried out at the following. In “Theoretical basis and research problem”section describes the theoretical basis and problem formulation; in “Theoretical deduction” section explores changing population changing law of urban agglomeration; in Empirical implementation” section conducts empirical research; in Discussion” section is research discussion; in Conclusion” section draws the research conclusion of this paper.

## Theoretical basis and research problem

### Theoretical basis

Traditional economic geography theory shows that location choice affects the spatial distribution of population. The choice of agricultural location leads to a circular crop planting. Especially, crop prices and land costs are positively correlated with proximity to the city center, thus maintaining the overall utility balance^30^. Industrial enterprises choose the location where labor and transportation costs are the least to maximize the utility^31^. In pursuing profit maximization, business and service industries have formed a center-periphery structure^32^. These theories demonstrate individuals migrate among different cities to obtain higher net utility, including wages, the quality of life, the level of medical care, and education.

Many scholars have studied the spatial distribution of the population by location selection based on traditional theories. One scholar pointed out that location selection is essential in causing population migration. A large number of people gathering will increase the cohesion and attractiveness of the region, causing more people to migrate there generate positive feedback on the flow of people^26^. Scholars found that immigrants with higher education levels are more likely to stay in the core area, provide more excellent value to the site, and further attract the inflow of talent^33^, the influx of immigrants hurts the land utilization and land cover have an impact^34^. The repeated transfer of population to core areas will lead to overpopulation, which will cause great pressure on resources and the environment^35^. Each stage of population location selection will cause changes in the spatial distribution of the population^34^. Individual location selection leads to different urban population scales. Especially in the early stage of urban agglomerations, the population size of central cities grew more prominently than that of surrounding cities. At the mature stage of urban agglomerations, the population size of surrounding cities increased more significantly than that of central cities^26,36^.

**Figure 1:**
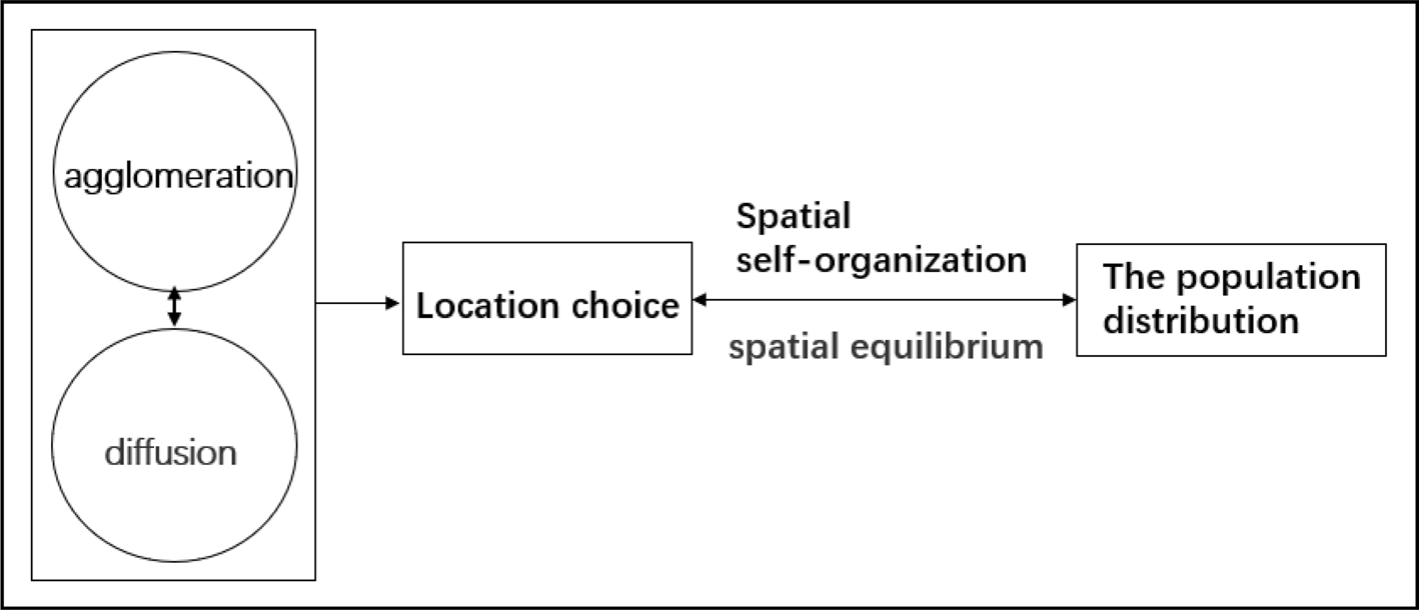
Influencing mechanism of population distribution.

Urban self-organization and spatial equilibrium affect the spatial distribution of the population. Under the spatial self-organization mechanism and spatial equilibrium, enterprises and individuals migrate among cities to pursue higher net utility values. This migration will not stop until the net utility value of cities is approximately equal. In this state, individuals and enterprises had no desire to migrate, and urban population spatial distribution achieved equilibrium^15,37^. That is to say, a common mechanism guides the development between cities. Through the analysis of urban development and urban form, some scholars have found that the expansion of cities is similar, and they follow the influence of spatial self-organization and spatial equilibrium and develop towards a more homogeneous urban form^8^. Some scholars have also put forward other evidence, they found that the existence and development of the city is the result of the interaction of centripetal force and centrifugal force, which can reflect that the internal system of the town is affected by the self-organization and spatial equilibrium of the city^38^. Other scholars describe the changes in the internal system of cities as intense social interaction, and the diverse social and economic activities are the embodiment of spatial self-organization and spatial equilibrium^12^. The influencing mechanism of population distribution is shown in Fig. 1.

The mechanism of aggregation and diffusion is a deeper reason that affects the spatial distribution of the population^25^. The mechanism of population agglomeration drives the spatial growth of urban agglomerations. The spatial differences and imbalances in economic development between cities have become more prominent, forming the "Matthew Effect", which affects the spatial distribution of population. The population diffusion mechanism promotes the relative spatial balance of resources, economy, factors, and enterprises, conducive to narrowing the gap in urban economic levels within urban agglomerations and promoting collaborative economic development. Existing research points out that agglomeration and diffusion are the primary stimuli and regional development processes. Through the agglomeration and diffusion of various elements such as technology, capital, talents, and information, an interactive relationship between urban and rural areas is established, promoting changes in the spatial distribution of population^25,39^.

It is also found that the interaction between agglomeration and diffusion is an important mechanism involved in the urban population spatial distribution^25^. The aggregation and diffusion mechanisms perform differently at each stage of urban agglomeration development. The agglomeration mechanism is more evident in the early stages of urban agglomeration development. At this time, the central city has rich in natural resources and attracts people to gather. The diffusion mechanism becomes more effective in the mature period of urban agglomerations. Rising costs in central cities drive people out. Also, the agglomeration mechanism and diffusion mechanism have a certain inertia. Once aggregation (or diffusion) occurs, it will continue to develop in the inherent direction, with changes in content, scale, level, and speed. When agglomeration diseconomy (or diffusion diseconomy) occurs, the trend of accumulation (or distribution) can be curbed—the interaction changes from aggregation-based to diffusion-based (or from diffusion-based to aggregation-based). However, the original accumulation (or distribution) will not disappear, and it will still exist in the form of diffusion (or aggregation).

### Problems

**Figure 2:**
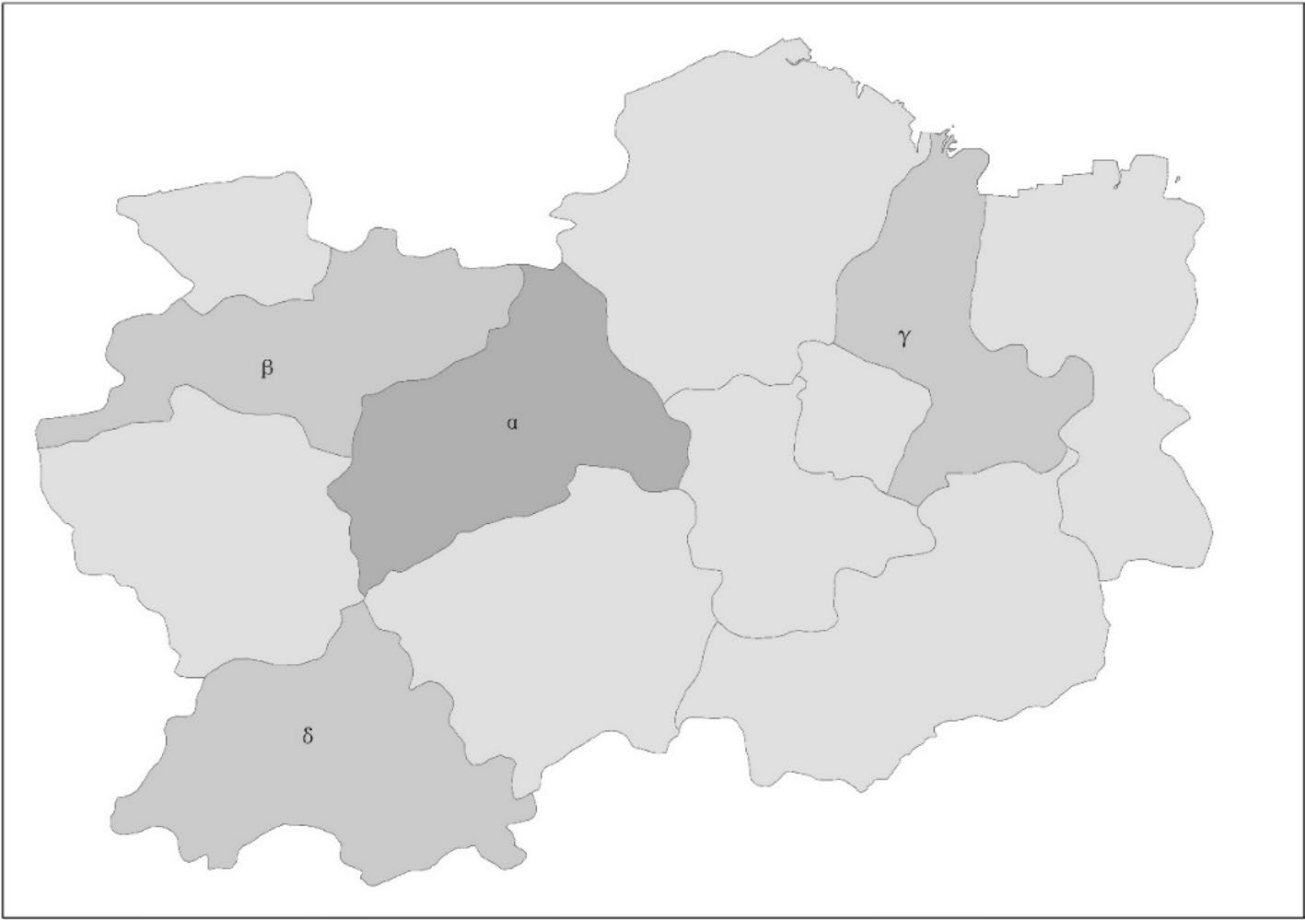
The virtual map of urban agglomeration. (ArcGIS10.2,URL: https://developers.arcgis.com/).

We set up a virtual urban agglomeration to derive the law of population size distribution of urban agglomeration. As shown in Fig. 2, the urban virtual agglomeration includes ***m*** central cities and ***n*** peripheral cities according to their level of economic development (***m*** < ***n***). It is assumed that housing costs and living costs are the same in central and peripheral cities in the early period of urban agglomeration, in the mature stage of urban agglomeration, the housing cost of central cities is much higher than in surrounding cities, and the living cost is the same^6,40^.

**Figure 3:**
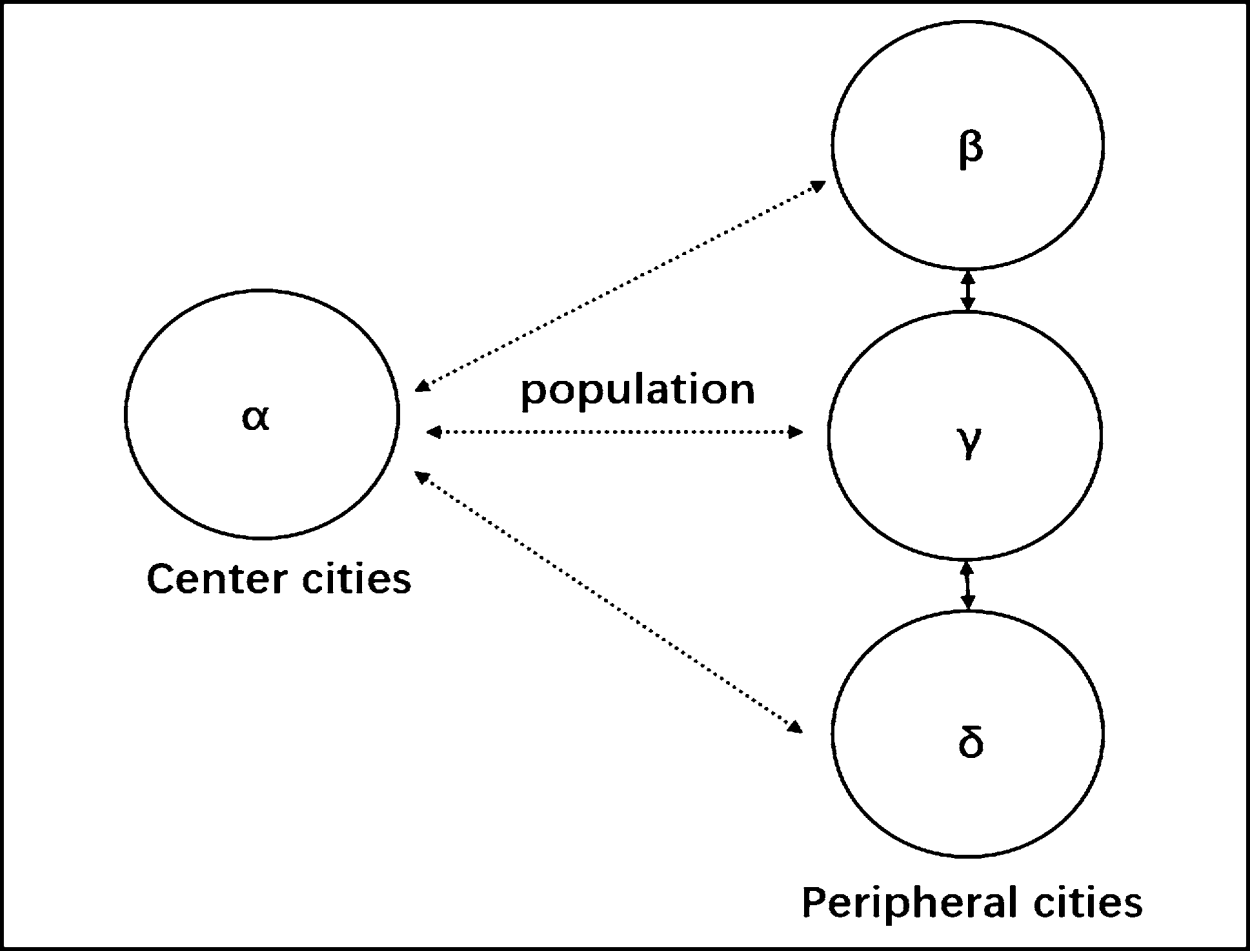
Population migrates among cities.

The ***α*** city of the central city and the ***β***, ***γ***, ***δ*** cities of the peripheral cities are selected, as shown in Fig. 3. Individuals and companies move between cities to maximize their utility. Population flows occur between central cities, peripheral cities, and central and peripheral cities. Urban agglomerations achieve spatial population balance through population flow.

We used the following assumptions in the reasoning process to deduce the law of spatial distribution of urban population.

1. A rational person pursues the maximization of net income. Specifically, we only considered economic factors in the process of individuals selecting locations. This assumption simplifies the factors that affect the location selection of the population and ignores the influence of other factors (e.g. behavioral characteristics).
2. The choice of population location is adjusted rapidly with the adjustment of population size. We ignored the influence of population density and built-up area on location selection.
3. The cost of living in different cities in the same urban agglomeration is the same. In other words, the population location factors that except income and housing factors in the same urban agglomeration should be homogenized.
4. The housing cost of cities in the urban agglomeration is positively correlated with development. The higher the level of urban development, the higher the cost of housing. This assumption simplifies the difference in housing costs caused by cities at different development levels. It is conducive to analyzing population location selection from housing cost differences.

## Theoretical deduction

### The changing trend of spatial population distribution in urban agglomerations

We took any central city ***α*** and any peripheral city ***β*** to analyze the spatial distribution characteristics of urban agglomerations under different development levels. The meaning of each letter is as follows: ***E*** is the net utility value, ***W*** is the per capita income level, ***C*** is the cost, ${\varvec{C}}_{1}$ is the cost of living, ${\varvec{C}}_{2}$ is the cost of housing, ***s*** is the initial state, ***r*** is the critical state, and ***l*** is the final state.

In the initial state of city agglomeration development, the per capita income level of central cities is higher than that of peripheral cities^21^, and the housing costs and living costs of central cities are the same as those of peripheral towns^40–42^.

1 $$E_{\alpha s} = W_{\alpha s} - C_{1\alpha s} - C_{2\alpha s}$$

2 $$E_{\beta s} = W_{\beta s} - C_{1\beta s} - C_{2\beta s}$$

Under the initial state of city agglomeration development, $W_{\alpha s}$ > $W_{\beta s}$, $C_{1\alpha s}$ = $C_{1\beta s}$, $C_{2\alpha s}$ = $C_{2\beta s}$, so $E_{\alpha s}$ > $E_{\beta s}$. The net utility value of central cities is greater than that of peripheral cities. From condition 1, the population gathers in the central town^38,43^.

In mature urban agglomerations, the per capita income of central cities is higher than that of peripheral cities. The cost of housing in central cities is much greater than the cost of living in surrounding cities, and the cost of living in central cities is the same as that of the surrounding towns^40,42^.

3 $$E_{\alpha l} = W_{\alpha l} - C_{1\alpha l} - C_{2\alpha l}$$

4 $$E_{\beta l} = W_{\beta l} - C_{1\beta l} - C_{2\beta l}$$

In the final state,$W_{\alpha l}$ > $W_{\beta l}$, $C_{1\alpha l} = C_{1\beta l}$, $C_{2\alpha l}$ >  > $C_{2\beta l}$, so $E_{\alpha l}$ < $E_{\beta l}$, the conclusion is that in the later period of the city agglomeration, the net utility value of peripheral cities is greater than that of central cities. Thus, according to Condition 1, the population began to gather in peripheral cities^36,44^. The above reasoning process leads to the first conclusion:

Conclusion 1: In the early stage of urban agglomeration development, the population tends to gather in central cities. In the mature period of urban agglomeration, the population is concentrated in peripheral cities.

### The changing trend of urban agglomeration population gravity center

From the above chapter, cities within urban agglomeration will experience varying population migration due to different development levels. We choose the population center of gravity indicator to visually express the population flow changes. The population center of gravity refers to the point at which the moment of population distribution on the spatial plane reaches equilibrium, usually used to measure the equilibrium of population distribution in the region^45^. By studying the movement trajectory of the population center of gravity, we explain the characteristics and causes of the spatial change of population distribution , and provide a decision-making basis for formulating population development policies and social and economic development plans. We took the permanent population data of municipal districts as weights and weighted the spatial units to get the population center of gravity. The calculation method is as follows:

5 $${\text{X}} = \frac{{\mathop \sum \nolimits_{i = 1}^{n} M_{i} X_{i} }}{{\mathop \sum \nolimits_{i = 1}^{n} M_{i} }}{,}\,\,{\text{Y}} = \frac{{\mathop \sum \nolimits_{i = 1}^{n} M_{i} Y_{i} }}{{\mathop \sum \nolimits_{i = 1}^{n} M_{i} }}$$

where, $X_{i}$ and $Y_{i}$ represent the longitude and latitude coordinates of the ith spatial unit, respectively, and $M_{i}$ represents the population size of a city in the spatial unit.

It is assumed that there is a central city and a peripheral city in the urban agglomeration, where the location of the central city is ($X_{1}$, $Y_{1}$) and the population size is $M_{1 }$.The location of the peripheral cities is ($X_{2}$, $Y_{2}$), and the population size is $M_{2}$.The population center of gravity position of this urban agglomeration is (X, Y).

1. To judge the change in the population center of gravity in the early urban agglomeration, we assume that the population of the central city $M_{2}$ remains unchanged, find the derivation of $M_{1}$.${\text{ M}}_{1} > 0,$ ${\text{M}}_{2}$ > 0.

1. Longitude.

$$\begin{aligned} \frac{{d_{x} }}{{d_{M1} }} & = \frac{{X_{1} \left( {{\text{M}}_{1} + {\text{M}}_{2} } \right) - (M_{1} X_{1} + M_{2} X_{2} )}}{{\left( {{\text{M}}_{1} + {\text{M}}_{2} } \right)^{2} }} \\ & = \frac{{M_{2} \left( {{\text{X}}_{1} - {\text{X}}_{2} } \right)}}{{\left( {{\text{M}}_{1} + {\text{M}}_{2} } \right)^{2} }} \\ \end{aligned}$$

If ${\text{X}}_{1}$ > ${\text{X}}_{2}$ (the central city is to the east of the peripheral city), $\frac{{{\text{d}}_{{\text{x}}} }}{{{\text{d}}_{{{\text{M}}2}} }}$ > 0. The longitude of the population gravity center increases (moving eastward), that is, toward the central cities. If ${\text{X}}_{1}$ < ${\text{X}}_{2}$ (the central city is to the west of the peripheral city), $\frac{{{\text{d}}_{{\text{x}}} }}{{{\text{d}}_{{{\text{M}}2}} }}$ < 0. The longitude of the population gravity center decrease (moving westward), that is, toward the central cities.

In conclusion, when the peripheral city population remains unchanged, the population size of the central city increases (when the population size of the central city increases more than that of the peripheral city increases), the population gravity center's longitude moves toward the central city population.

(2) latitude

$$\begin{aligned} \frac{{d_{y} }}{{d_{M1} }} & = \frac{{Y_{1} \left( {{\text{M}}_{1} + {\text{M}}_{2} } \right) - (M_{1} Y_{1} + M_{2} Y_{2} )}}{{\left( {{\text{M}}_{1} + {\text{M}}_{2} } \right)^{2} }} \\ & = \frac{{M_{2} \left( {{\text{Y}}_{1} - {\text{Y}}_{2} } \right)}}{{\left( {{\text{M}}_{1} + {\text{M}}_{2} } \right)^{2} }} \\ \end{aligned}$$

If ${\text{Y}}_{1}$ > ${\text{Y}}_{2}$ (the central city is to the north of the peripheral city), $\frac{{{\text{d}}_{{\text{Y}}} }}{{{\text{d}}_{{{\text{M}}2}} }}$ > 0. The latitude of the population gravity center increases (moving north), that is, toward the central cities. If ${\text{Y}}_{1}$ < ${\text{Y}}_{2}$ (the central city is to the south of the outer city), $\frac{{{\text{d}}_{{\text{Y}}} }}{{{\text{d}}_{{{\text{M}}2}} }}$ < 0. The latitude of the population gravity center decrease (moving south), that is, toward the central cities.

In conclusion, when the population of peripheral cities remains unchanged, but the population size of central cities increases (or when the population size of central cities increases more than that of peripheral cities), the population gravity center moves to the central cities in latitude.

2. To judge the change of population center of gravity in the mature urban agglomeration, we assume that the population of the central city $M_{1}$ remains unchanged, find the derivation of ${ }M_{2} \cdot M_{1} > 0,$ $M_{2}$ > 0.

1. Longitude.

$$\begin{aligned} \frac{{{\varvec{d}}_{{\varvec{x}}} }}{{{\varvec{d}}_{{{\varvec{M}}2}} }} & = \frac{{X_{2} \left( {{\text{M}}_{1} + {\text{M}}_{2} } \right) - (M_{1} X_{1} + M_{2} X_{2} )}}{{\left( {{\text{M}}_{1} + {\text{M}}_{2} } \right)^{2} }} \\ & = \frac{{{\varvec{M}}_{1} \left( {{\text{X}}_{2} - {\text{X}}_{1} } \right)}}{{\left( {{\text{M}}_{1} + {\text{M}}_{2} } \right)^{2} }} \\ \end{aligned}$$.

If ${\text{X}}_{1}$ > ${\text{X}}_{2}$ (the central city is to the east of the outer city), $\frac{{{\text{d}}_{{\text{x}}} }}{{{\text{d}}_{{{\text{M}}2}} }}$ < 0. The longitude of the population gravity center decrease (moving westward), that is, toward the peripheral cities. If ${\text{X}}_{1}$ < ${\text{X}}_{2}$ (the central city is to the west of the outer city), $\frac{{{\text{d}}_{{\text{x}}} }}{{{\text{d}}_{{{\text{M}}2}} }}$ > 0. The longitude of the population center of gravity increase (moving eastward), that is, toward the peripheral cities.

To sum up, when the population of the central city remains unchanged, and the population size of the peripheral city increases (or when the increase in the population size of the surrounding cities is more significant than the increase in the population size of the central cities), the longitude of the population gravity center moves to the peripheral cities.

(2) latitude

$$\begin{aligned} \frac{{{\varvec{d}}_{{\varvec{y}}} }}{{{\varvec{d}}_{{{\varvec{M}}1}} }} & = \frac{{{\varvec{Y}}_{2} \left( {{\text{M}}_{1} + {\text{M}}_{2} } \right) - ({\varvec{M}}_{1} {\varvec{Y}}_{1} + {\varvec{M}}_{2} {\varvec{Y}}_{2} )}}{{\left( {{\text{M}}_{1} + {\text{M}}_{2} } \right)^{2} }} \\ & = \frac{{{\varvec{M}}_{1} \left( {{\text{Y}}_{2} - {\text{Y}}_{1} } \right)}}{{\left( {{\text{M}}_{1} + {\text{M}}_{2} } \right)^{2} }} \\ \end{aligned}$$

If $Y_{1}$ > $Y_{2}$ (the central city is to the north of the outer city), $\frac{{{\text{d}}_{{\text{Y}}} }}{{{\text{d}}_{{{\text{M}}2}} }}$ < 0. The center of gravity of the population decreases latitude (moving southward), moving toward the outer cities. If $Y_{1}$ < $Y_{2}$ (the central city is to the south of the outer city), $\frac{{{\text{d}}_{{\text{Y}}} }}{{{\text{d}}_{{{\text{M}}2}} }}$ > 0, the center of gravity of the population increases latitude (moving northward), moving toward the outer cities.

In conclusion, when the population size of the central city remains unchanged, and the population size of the peripheral city increases (the increase of the population size of the peripheral city is greater than that of the central city), the latitude of the population gravity center moves to the peripheral cities. The above reasoning process leads to the second conclusion:

Conclusion 2: The latitude and longitude changes of the population gravity center reveal the characteristics of the spatial distribution of the population in the urban agglomeration. In the initial stage of urban agglomerations, the population size of the central city increased more than that of the surrounding cities, and the population gravity center shifted to the central city. In the mature period of urban agglomeration development, the population growth of surrounding cities is greater than that of central cities, and the population gravity center shifts to surrounding cities.

### Changing in equilibrium degree of spatial population distribution in urban agglomerations

Based on four hypotheses and existing research, this paper demonstrated the equilibrium changes of the spatial population distribution of urban agglomerations at different development levels. In the initial state of urban agglomeration development, the productivity level is low, the economy is underdeveloped, the urbanization level is relatively low, and the city's population is generally in a low-level equilibrium state^46^. Some cities develop rapidly through external stimulation or long-term accumulation^47^. Various favorable factors have driven the population to shift to advantageous cities, and central cities have gradually formed^48,49^. The formation of central cities in urban agglomerations broke the balance of the original urban agglomeration population spatial structure. Specifically, the population gap between central and other cities continues to widen. The concentration of population in central cities has caused congestion in central cities, further increasing land costs. A series of urban problems have emerged, ultimately reducing the net utility value of central cities^6^. The net utility value of the central city is gradually more minor than the net utility value of the peripheral cities. According to Condition 1, the population begins to migrate to the surrounding towns, the population gap between the central city and the peripheral cities is gradually reduced, and the spatial population distribution of the urban agglomeration tends to be balanced.

Population spatial balance in urban agglomerations is a complex process of urban self-regulation caused by people's pursuit of utility maximization^20^. The direction of population migration is related to the relative gap between cities at different development levels in urban agglomerations. The population flows in cities with high development levels and immense net utility value, ultimately affecting the spatial equilibrium of people in urban areas agglomerations^8,26^. The above reasoning process leads to the third conclusion:

Conclusion 3: In the early stage of urban agglomeration development, the degree of spatial population equilibrium gradually decreases with the development of urban agglomeration. When urban agglomerations reach the mature stage, the degree of spatial equilibrium of the population increases gradually with the development of urban agglomerations.

## Empirical implementation

### Sample selection

We choose the Beijing–Tianjin–Hebei Urban Agglomeration (denoted as BTHUA) population data from 2005 to 2018 as the research object. The BTHUA comprises 13 cities, including Beijing, Tianjin, Langfang, Tangshan, Qinhuangdao, Chengde, Zhangjiakou, Baoding, Shijiazhuang, Xingtai, Handan, Hengshui, and Cangzhou. Among them, the central city is Beijing.

We chose BTHUA as our study object is suitable. BTHUA is one of the critical population agglomerations in China. By the end of 2018, 112.7 million permanent residents accounted for 8.08 percent of the country's total population. A large sample size can avoid bias caused by a small sample size. As the capital of China, Beijing is a political, cultural, and economic center that has attracted a large number of migrants in recent decades. The ability of central and peripheral cities is significantly different, which can better reflect the population flow in the development of urban agglomerations^35^. The BTHUA has improved the quality of economic growth and the overall development level of the urban agglomeration through cooperation and complementarity, representing a certain degree.

### Data sources

Since the urban population is not easy to obtain, we use the resident population of the prefecture-level municipal districts to replace the urban population for calculation. This paper chooses the permanent population as the research object, mainly referring to the registered and floating folks who have lived in cities for more than half a year^50–52^. Permanent resident population overcomes population mobility to some extent and can fully reflect the spatial characteristics of the people in the regular operation of a city^53^. There are no data on the permanent population in the municipal area, but data on the municipal area's GDP and per capita GDP. The permanent urban population is obtained through the quotient of the above two data. This article collects the GDP and per capita GDP data of 13 cities in China from 2005 to 2018 in the China City Statistical Yearbook.

### Empirical tests and results

#### The changing trend of spatial population distribution in BTHUA

We collected the population data of each city in the urban agglomeration to analyze the changes in the population size of each level, as shown in Table 1. The total population of the BTHUA continues to grow, and the population growth rate is relatively stable in the short to medium term. The population size of central and peripheral cities also increased, but the proportional trend changed. Specifically, the proportion of the population in the central cities of the urban agglomeration dropped from 41.27 to 35.94%, and the population size of the outer towns increased the proportion of urban agglomerations from 58.73 to 64.06%.

**Table 1:** The population size of each type of city in the BTHUA (more detailed data are available in supplementary materials).

Year	Total population size (ten thousand people)	Central city (ten thousand people)	Peripheral cities (ten thousand people)	The ratio of major cities (%)	The percentage of peripheral cities (%)
2005	3496.767	1443.241	2053.526	41.27%	58.73%
2006	3487.686	1486.762	2000.924	42.63%	57.37%
2007	3699.009	1533.45	2165.559	41.46%	58.54%
2008	3866.576	1590.049	2276.527	41.12%	58.88%
2009	3811.187	1650.491	2160.696	43.31%	56.69%
2010	4189.215	1781.544	2407.672	42.53%	57.47%
2011	4586.717	1911.51	2675.207	41.67%	58.33%
2012	4677.826	1964.889	2712.937	42.00%	58.00%
2013	4839.318	2009.39	2829.928	41.52%	58.48%
2014	5083.074	2053.893	3029.181	40.41%	59.59%
2015	5641.707	2161.055	3480.652	38.30%	61.70%
2016	5925.251	2171.706	3753.545	36.65%	63.35%
2017	5997.658	2171.802	3825.856	36.21%	63.79%
2018	6016.714	2162.454	3854.261	35.94%	64.06%

#### The changing trend of the population gravity center at BTHUA

**Figure 4:**
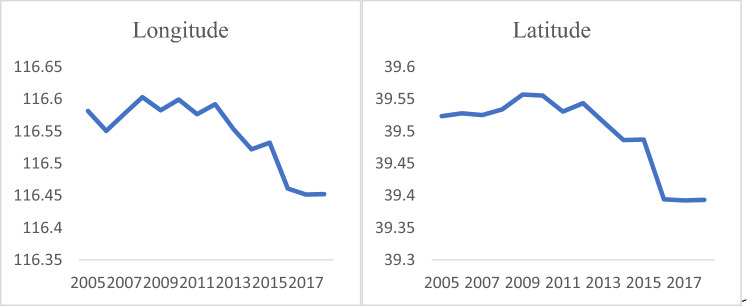
Longitude and latitude changes of population gravity center in BTHUA.

We took BTHUA as an example to verify the results of the derivation of the population gravity center in 3.2 simulated urban agglomerations. We calculated the latitude and longitude of the BTHUA population center of gravity from 2005 to 2018 according to the population gravity center formula in Eq. (5). The result is shown in Fig. 4. The latitude and longitude of the BTHAU population center of gravity showed an overall downward trend. Specifically, the latitude and longitude changes were manifested as fluctuations from 2005 to 2012 and a decline from 2012 to 2018.

**Figure 5:**
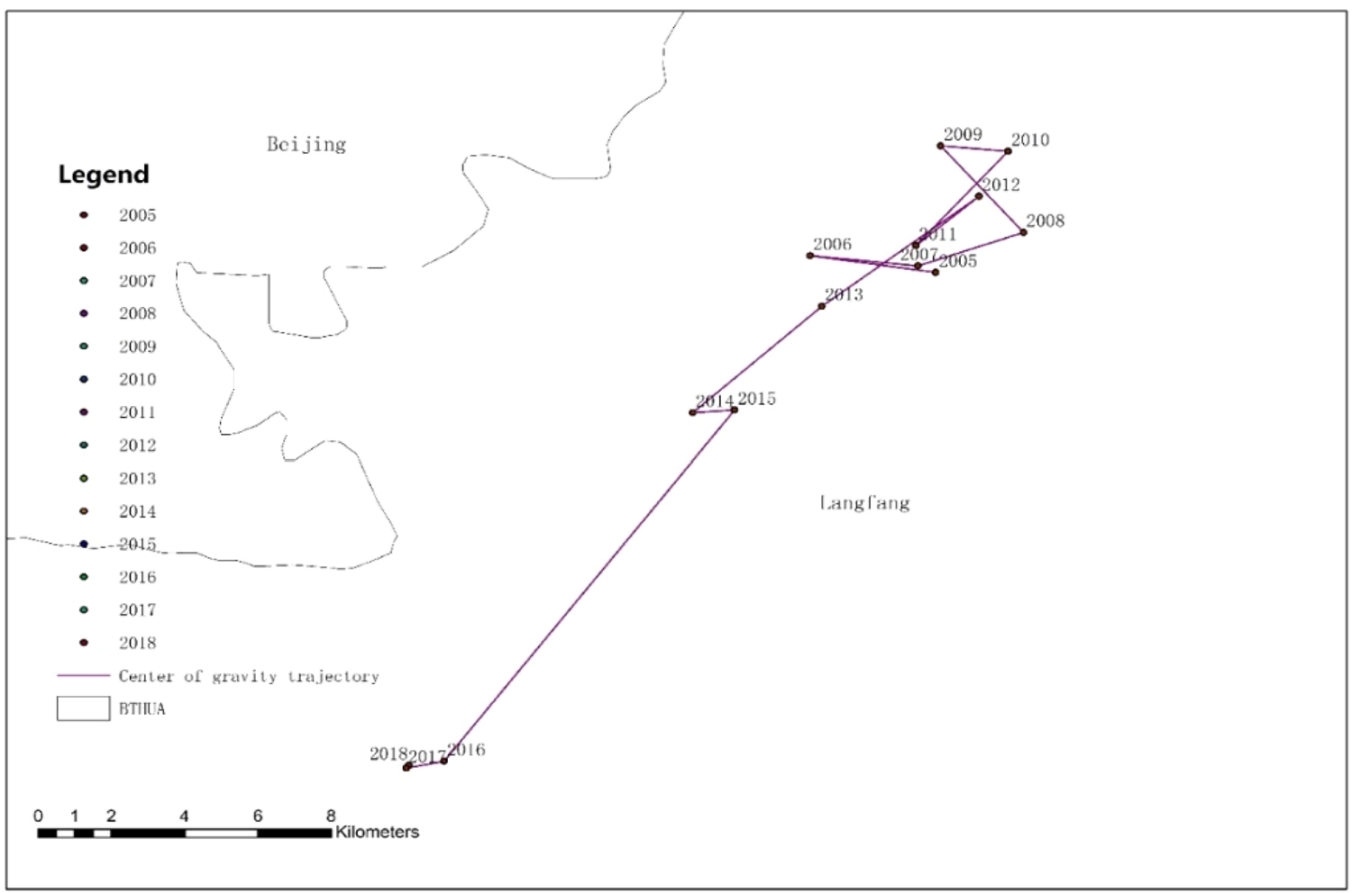
The change tracking of the population gravity center in BTHUA. It mainly shows the migration of the BTHUA population gravity center in the past 14 years (more details in the supplementary document).

**Figure 6:**
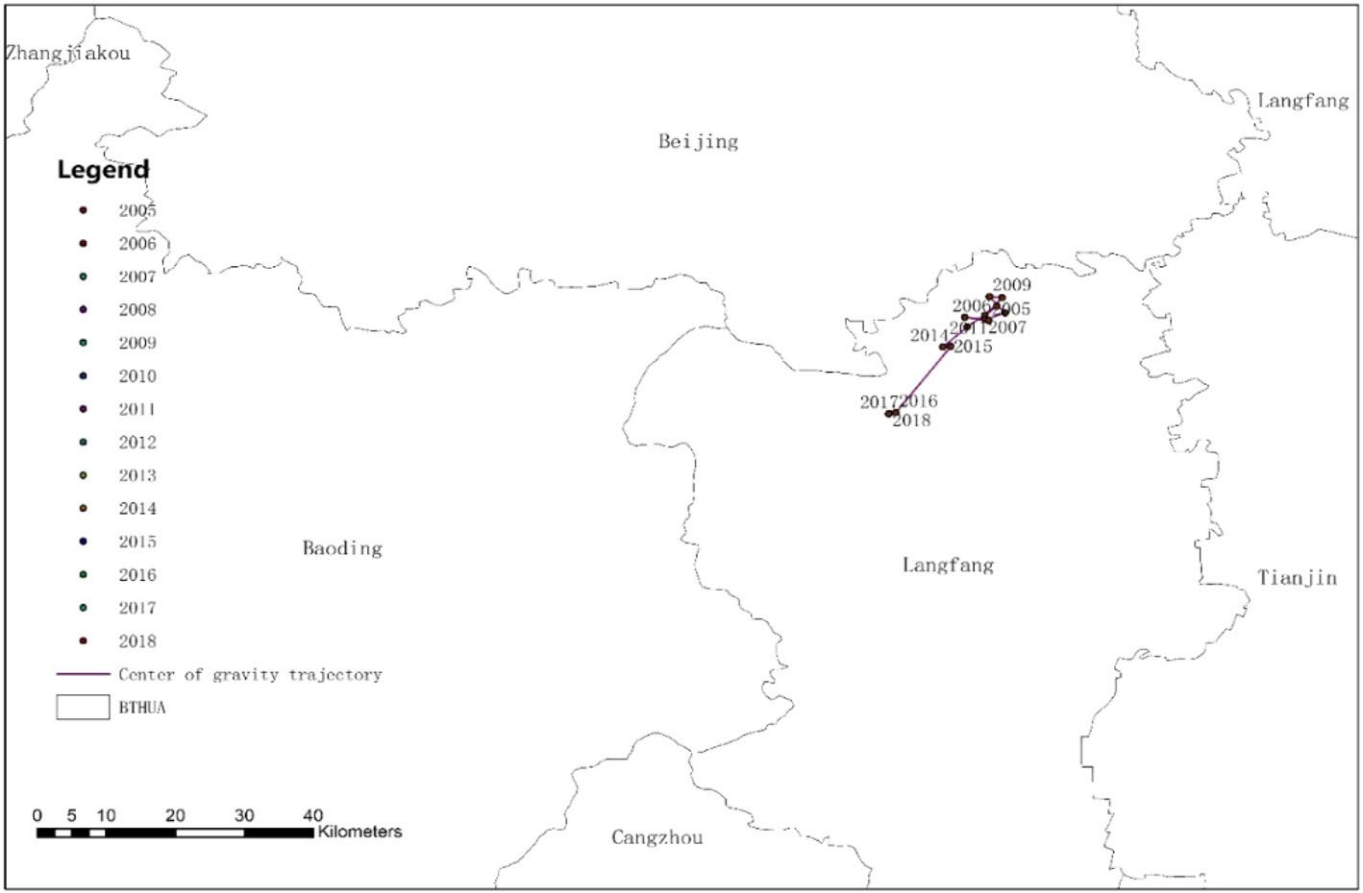
Study on the changing track of the population gravity center in BTHUA. It mainly shows the specific location of the population gravity center at BTHUA.

We used GIS technology to draw the BTHUA permanent population gravity center distribution map from 2005 to 2018 and visually describe the BTHUA population gravity center trend. We connected the annual population gravity center to form a trajectory of the population center of gravity change. From the migration of the population gravity center in Figs. 5 and 6, we can see that from 2005 to 2012, the population center of BTHUA was located in Langfang from 2005 to 2012; but BTHUA's population gravity center shifted to the central city. From 2012 to 2016, the population center of BTHUA moved to the southwest, and from 2016 to 2018, the center of population moved to the west, all are far away from the central city. To sum up, the population center of the BTHUA is to first migrate to the central cities and then to the peripheral cities.

#### The equilibrium change trend of spatial population distribution in BTHUA

**Figure 7:**
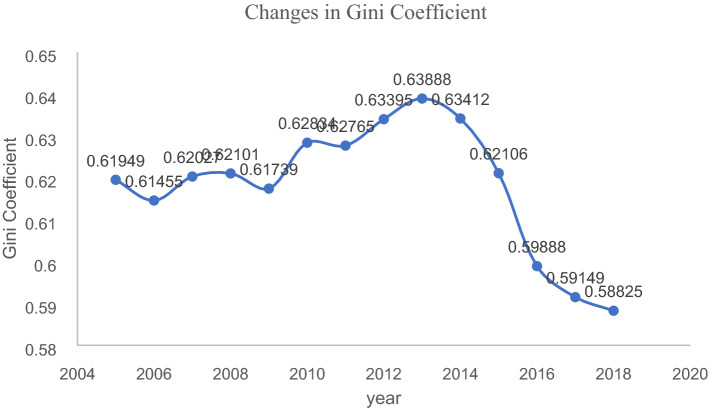
Changes in Gini Coefficient of population size in BTHUA.

We calculated the population Gini coefficient of BTHUA to judge the balanced change in the BTHUA population spatial distribution. The population Gini coefficient describes the spatial distribution pattern of population size, profoundly explores the law of population size spatial distribution, creates a suitable environment for the healthy growth of population size, and promotes the rational public allocation of facility resources. The population Gini coefficient is calculated as follows^54^:

6 $${\mathbf{G}} = \frac{{\varvec{T}}}{{2{\varvec{S}}\left( {{\varvec{n}} - 1} \right)}}$$

where ***n*** indicates the number of cities contained in the urban agglomerations, ***S*** is the total urban population of the whole urban agglomerations, and ***T*** is the sum of the absolute value of the difference in population size between cities in the urban agglomeration. ***G*** ∈ ^23^, the closer ***G*** is to 0, the more dispersed the urban population distribution and the lower the spatial aggregation of urban agglomerations, and the closer ***G*** is to 1, the more concentrated the urban scale^54^. We calculated the Gini coefficient using the BHTAU data, and the results are shown in Fig. 7.

We constructed a regression equation of the Gini coefficient over time (years), which vividly reflected the equilibrium changes in the population size of urban agglomerations. As shown in Fig. 7, the Gini coefficient of the BTHUA population size shows an "inverted U-shaped" change. From 2005 to 2013, the Gini coefficient fluctuates wildly, but the overall trend increases. The general direction of the Gini coefficient from 2013 to 2018 is to decrease. Specifically, the Gini coefficient of population size declines rapidly from 2013 to 2016 and slowly falls from 2016 to 2018.

The degree of population equilibrium is related to the population Gini coefficient. When the population gravity center migrates to the central city, the population size gap is more prominent, that is, the degree of population balance is poor, and the Gini coefficient increases at this time; when the population gravity center migrates to peripheral cities, the population size gap between cities becomes smaller, that is, the population balance degree is higher, and the Gini coefficient becomes smaller. Specifically, from 2005 to 2012, the population center of gravity gradually approached BTHUA's central city, Beijing, indicating that the population of the central city has increased dramatically, the population gap between cities in the urban agglomeration has increased, the degree of equilibrium is low, and the Gini coefficient is relatively large. From 2013 to 2018, the population center of BTHUA moved to the southwest and gradually moved away from the central city. At this stage, the population gap has narrowed, the population distribution of urban agglomerations has tended to be balanced, and the Gini coefficient has declined.

## Discussion

### The urban population of different development levels in urban agglomerations has different trends

As is shown in Table 1, the population size of the central city has experienced a first increase and then a decline. In contrast, the population size of the peripheral cities has experienced an early fall, and a later rise and conclusion 1 has been verified. The results of this research are consistent with Egidi, who found that the size of the urban population is related to the speed of population migration by establishing a theoretical model of urban growth^17^. It also supplements Kim's research conclusions, who took the urban population of the United States as an example and found that the population increased both in suburbs and central. However, the growth trends had significant differences^55^. This law is also verified by Polinesi, who used Greek cities as an example to establish a partial regression model and found that population growth is internally affected by cities with different levels of development^19^.

The difference from previous studies is that we deduce the changing law of the spatial distribution of population in urban agglomerations to demonstrate the factors that affect this law. The factors affecting the spatial distribution of population include public service factors such as education and medical care, social factors such as interpersonal relationships, social culture, climate, temperature, and other natural environmental factors, etc., which affect personal income and costs. If a city's per capita net income is higher than its neighbors, people will flock to that city. Therefore, increasing the net utility of residents in surrounding cities is an effective measure to alleviate the population problem in large cities. The influencing factors mentioned above are the only way to increase the net utility value of residents, such as raising wages, lowering the cost of living, improving infrastructure, improving education, etc.

This paper judged the direction of population migration through theoretical derivation and improved the content of existing literature on population migration. Taking cities within urban agglomerations as the unit, this paper also deduces the population migration and its change process in each stage of urban agglomerations life cycle, which is beneficial for the government to implement different planning and guidance for urban agglomerations with varying levels of development and improve decision-making efficiency.

### The population center of gravity in the urban agglomeration first moves to the central city and then to the outer cities

As is shown in Figs. 5 and 6, the population centers of BTHAU from 2005 to 2018 are connected in chronological order to establish the spatial relationship of cities between urban agglomerations. The results show that the population center first shifts to central and peripheral cities. This study not only validates Conclusion 2 but also validates the studies of other scholars. For example, This conclusion verifies that You ‘s proposal of population migration to large cities with a high degree of urbanization and commercialization^33^. And this conclusion also verifies Lemoine-Rodriguez‘s decision that in the process of sustainable urban development, surrounding cities show the most remarkable vitality in the expansion and change of urban morphology and become the key to sustainable urban development (Lemoine-Rodríguez^8^).Urban agglomerations at different stages of development are affected by policy, environment, economy, and other factors, which lead to varying trends of population change. It's worth saying that our demographic shift was inspired by the work of Ivan Turok, who divides the development process of urban agglomerations into two stages, one in the early development stage of the urban agglomeration, and the other is the maturity of the urban agglomeration. Our research has also confirmed that the development of urban agglomerations has phases and has certain laws^6^.

It can be seen from the above description that BTHAU is currently in the mature stage of urban agglomerations, and the central city is driving the development of surrounding cities. This discovery will help implement the central City and BTHAU's mid—and long-term development plans under the new development model and strive to achieve the tenth Five-year Plan and the 2035 development goals. Urban agglomerations have the homogeneity of development, and the development process of BHTUA has reference significance for other urban agglomerations in the early stages of development.

### The spatial distribution of population in urban agglomerations tends to be balanced and then unbalanced

As shown in Fig. 7, the population Gini coefficient of the BTHUA exhibits an "inverted U"-shaped change. The inverted "U" curve shows that as the level of urban development increases, the spatial distribution of the population presents an imbalance first and then tends to the trend of balance. This result verifies the rationality of Conclusion3. This conclusion is similar to Williamson's inverted U-shaped theory. Williamson’' 's inverted U-shaped curve reveals the relationship between the degree of regional imbalance and the level of economic development. As the economy grows, the income gap first widens and then narrows^56^. Our research reveals the relationship between population imbalance and urban development. As the level of urban development increases, the population Gini coefficient shows a change that first expands and then shrinks. Williamson and our inverted "U" curves have in common that they both represent the relationship between the degree of equilibrium of an indicator and economic development. The longitude and latitude changes of the population center in Fig. 4 are also inverted "U" shapes, corresponding to the changing trend of the Gini coefficient, which also shows the reliability of this study to a certain extent.

According to 5.2, we know that BTHAU is in the mature stage of urban agglomeration development, and we guessed that it is the function of self-organization, aggregation, and diffusion mechanism within urban agglomeration, which promotes cities in urban agglomeration to maximize their utility and achieve sustainable development of urban agglomeration population. The above statement also reminds the government and policymakers that urban agglomerations have internal mechanisms to promote their development. We should guide the development of urban agglomerations based on respecting objective facts.

### Policy implication

An urban agglomeration is a semi-organic system that attracts and diffuses capital and information for development and expansion in urban agglomerations, combined population, resources, environment, society, and economy^26^. The government plays a vital role in the development of urban clusters. Under the government's guidance, the future development of the urban agglomeration will evolve into an integrated community of economy and destiny with integrated regional industrial layout, infrastructure construction, market construction, urban and rural planning and construction, environmental protection, and ecological construction. To achieve this high level of integration, the central city and peripheral cities will share a joint master plan, urban–rural development, transportation network, information flow, marketization, technological development, environmental protection, and remediation. Our research is oriented to various stages of urban agglomeration development, so we propose corresponding suggestions based on each step.

From the perspective of guiding the coordinated development of cities, when the overall economic development level of urban agglomerations is low, the government should vigorously develop central cities, improve the general level of central urban areas, encourage industrial upgrading and industrial transfer, and improve the development level of urban agglomerations; When the overall economic development level of the cluster is relatively high, the gap between cities is rather large. Policies should encourage central cities to give full play to their urban efficiency and development advantages, exert agglomeration and radiation effects, promote the development of surrounding cities, and narrow the gap between the centre and surrounding towns. From the perspective of spatial self-organization, each city in the urban agglomeration has a division of labor and cooperation with complementary advantages to promote the development of the entire urban agglomeration. The spatial population distribution of the urban agglomeration conforms to objective reality and follows certain laws. The government should reasonably guide the population distribution on respecting the law.

In the research process, there are still some problems that have not been solved, such as the built-up area is closely related to the population, but we ignore the impact of the built-up area on the people to simplify the model, which affects the actual value of the study to a certain extent. Specifically, rapid land use and land cover changes are rooted in urban population growth. As a land manager, the government should pay attention to the relationship between the population size of urban agglomerations and the area of built-up areas, grasp the development trend of the population size of urban agglomerations under the comprehensive framework of the economy, environment, and society, and formulate reasonable policies. In addition to ignoring the built-up area, the four assumptions also ignore public service factors such as education and medical care, social factors such as interpersonal relationships, social culture, climate, temperature and other natural environment factors, and population location factors. The influencing factors are also influenced by policies. Policy managers should identify the social macro environment and actively make judgments. In these relatively open and closely-connected places, the dynamic interaction of the population will promote the dynamic process of regional integration, resulting in social and cultural benefits and ensure that the value created by population diversity serves the wider public interest.

## Conclusion

The urban population scale has different development trends under different development levels. The population center of urban agglomeration migrates first to the central city and then to the peripheral city. In this process, the distribution of the urban population scale goes through two stages equilibrium to imbalance. This paper provides an empirical basis for the sustainable development of global urban agglomerations. Specifically, the spatial distribution of urban agglomerations is demonstrated from theoretical derivation and empirical analysis, which increases the universality of the study; Analyzing the change of population size in urban agglomeration from the perspective of time and space increases the completeness of the study. However, this paper still has the following deficiencies. In terms of theoretical derivation, the study of urban population spatial distribution based on the four premises has certain limitations. In the empirical analysis, we use the resident population of prefecture-level cities and municipal districts to replace the urban population. There is a particular uncertainty in the data, leading to deviations between the predicted and actual values. Using the population center of gravity method to judge the spatial distribution of the population may not be comprehensive enough. In the future, we can gradually relax the assumptions, increase the validity of data, and use more scientific methods to explore more rigorous changes in the population size of urban agglomerations (Supplementary Information).

## Supplementary Information

Supplementary Information.

## Data Availability

All data generated or analyzed during this study are included in this published article. These datasets were derived from the following public domain resources: http://tjj.beijing.gov.cn/, http://stats.tj.gov.cn/, http://tjj.hebei.gov.cn/.
